# Microbiota Assessment of Pediatric Simple and Complex Acute Appendicitis

**DOI:** 10.3390/medicina58091144

**Published:** 2022-08-23

**Authors:** Mohit Kakar, Aigars Reinis, Juta Kroica, Arnis Engelis, Renars Broks, Lasma Asare, Marelize Vermeulen, Simone Oliver Senica, Amulya Saxena, Aigars Petersons

**Affiliations:** 1 Department of Pediatric Surgery, Children’s Clinical University Hospital, LV-1004 Riga, Latvia; 2 Department of Pediatric Surgery, Riga Stradins University, LV-1007 Riga, Latvia; 3 Department of Biology and Microbiology, Riga Stradins University, LV-1007 Riga, Latvia; 4 Statistics Unit, Riga Stradins University, LV-1046 Riga, Latvia; 5 Faculty of Medicine, Riga Stradins University, LV-1007 Riga, Latvia; 6 Department of Pediatric Surgery, Chelsea Children’s Hospital, Chelsea and Westminster NHS Fdn Trust, Imperial College London, London SW10 9NH, UK

**Keywords:** simple and complex pediatric appendicitis, microbiota, *P. aeruginosa*, empirical antimicrobial treatment, antibacterial susceptibility

## Abstract

**Background and Objectives**. The aim of this study is to determine the prevailing microbiota in samples from pediatric patients with acute appendicitis, as well as evaluate the antibacterial sensitivity of the isolated microorganisms, comparing the data obtained with the clinic’s antibacterial therapy guidelines. **Materials and Methods**. The study group consisted of 93 patients between the ages of 7 and 18. All patients underwent a laparoscopic or conventional appendectomy. The children were hospitalized with signs and symptoms suggestive of acute appendicitis. Microbiological cultures from the appendix and abdominal cavity were collected intraoperatively. **Results**. *E. coli* was identified in most cases irrespective of the clinical presentation of acute appendicitis. Most strains were susceptible to ampicillin and amoxicillin/clavulanic acid. Five strains of *E. coli* produced extended spectrum beta-lactamase (ESBL). *Pseudomonas aeruginosa* (*P. aeruginosa*) was the second most commonly isolated causative agent. Furthermore, it was common in cases of acute complex appendicitis. Most strains of *P. aeruginosa* were resistant to amoxicillin/clavulanic acid, ertapenem, ampicillin and cefotaxime, yet were susceptible to ceftazidime. Regardless of the clinical presentation, the samples yielded mixed isolates. **Conclusion**. *E. coli* is the main causative agent of acute appendicitis in the pediatric population displaying susceptibility to various antibiotics. *P. aeruginosa* was more prevalent in cases of acute complex appendicitis. *P. aeruginosa* isolates were susceptible to ceftazidime; however, they were resistant to cefotaxime, which should, therefore, be removed from guidelines for empirical antibacterial treatment of acute appendicitis due to phenotypic resistance of *P. aeruginosa*. We recommend antibiotics with distinct implementation to avoid antibiotic resistance.

## 1. Introduction

The therapeutic plan for acute appendicitis has advanced in children to favor non-surgical antibacterial treatment over surgical treatment. Complicated appendicitis is the most common cause of intra-abdominal infections in children [1]. In order to decrease the risk of post-operative complications in cases of complicated appendicitis, such as wound infections and intraabdominal abscesses, antibiotics are included in the treatment protocols. However, there is a lack of unity with regards to the optimum choice for antibiotic regimens in cases of acute appendicitis in children. Moreover, the most suitable regimen can be subject to change depending on the geographical distribution of species of pathogenic and opportunistic pathogens as well as their antibacterial resistance. Thus, it is important to assess the etiopathology of pediatric appendicitis (simple and complex) clinically and analyze the antibacterial susceptibility of its causative agents [2,3].

The aim of this study is to determine the prevailing microbiota in samples from pediatric patients with acute appendicitis, as well as evaluate the antibacterial sensitivity of the isolated microorganisms, comparing the data obtained with the clinic’s antibacterial therapy guidelines. The results of the study showed that *P. aeruginosa* was more common in cases of acute complex appendicitis than in acute simple appendicitis. It was resistant to cefotaxime, which should therefore not be recommended for empirical antibacterial treatment of acute appendicitis. Antibiotics with different mechanisms of action should be used for treatment of acute complex appendicitis to avoid the development of antibiotic resistance.

## 2. Materials and Methods

A total of 93 patients (47 males, 46 females) between the ages of 7 to 18 were enrolled for this study due to limitations with the ethics approval. Patients were admitted to the Children’s Clinical University Hospital with complaints of acute abdominal pain in conjunction with signs and symptoms suggestive of appendicitis. Patients were screened pre-operatively to confirm or exclude this diagnosis. All patients underwent a laparoscopic or conventional appendectomy. Microbiological culture swabs from the appendix and peritoneal cavity were collected intraoperatively. The pediatric surgery team supervising the patients with appendicitis received written consent forms from the respective caregivers and assent from the patients if they were 13 years of age or older. The consent and assent were concerning to the research objective and methodology used for investigating the biological material [4,5]. Ethical approval: All procedures executed in this study involving human contenders were in accordance with the ethical standards of the institutional and/or the national research committee (Riga Stradins University, reference number: 21/27.04.2017.; as well as with the Children’s Clinical University Hospital, reference number: SP-37/2018.), the 1975 Helsinki declaration and its amendments were also included or other comparable standards of ethics. Data including patients’ age, sex, and medical history were poised prior to surgery.

Immediately following the appendectomy, the appendix was anatomized under barren circumstances, paired swab samples were taken from the intraluminal side of the appendix and an extra swab sample was taken from the submucosa. Specimens were placed in Amies transport medium for immediate transfer and subsequent bacterial culture [6]. They were cultured under aerobic and anaerobic conditions. Cultivation was performed on blood agar (Supplement, Oxoid, Hampshire, UK; Defibrinated Sheep blood—E&O laboratories limited, Falkirk, Scotland), MacConkey (Oxoid, UK) and trypticase soy (Oxid, UK) agar. Bacterial recognition was performed using the VITEK2 analyzer (Biomerieux, Auvergne-Rhone-Alpes, France).

Tests were conducted on antibacterial susceptibility, evaluations on the subsequent results were in accordance with recommendations from the European Committee on Antimicrobial Susceptibility Testing (EUCAST), more specifically ‘Clinical breakpoints and dosing of antibiotics’ (Version 8.0, 2020) [7]. Cultures that were cultivated overnight were suspended in physiological saline of up to 0.5 McFarland units (McFarland Densitometer DEN-1, Biosan, Riga, Latvia). The inoculation of the suspension was on Mueller-Hinton agar (Oxid, UK). Selected antibiotics were placed on the inoculated plates and included ceftazidime 10 µg, ampicillin 10 µg, cefotaxime 5 µg, meropenem 10 µg, imipenem 10 µg, amikacin 30 µg, gentamicin 10 µg, ciprofloxacin 5 µg, chloramphenicol 30 µg ertapenem 10 µg, amoxicillin/clavulanic acid 30 µg and piperacillin/tazobactam 36 µg (Liofilchem, Roseto degli Abruzzi, Italy). Plates were inoculated at a temperature of +35 ± 1 °C for 18 ± 2 h. According to the EUCAST standard, the double-disk synergy test (DDST) was used to detect extended spectrum beta-lactamase *E. coli* (ESBL). Results were assessed by measuring the zone of inhibition, and resistance was explained in accordance with the EUCAST breakpoints.

Statistical analysis was performed using Microsoft Excel 2016 (Microsoft, USA) and IBM SPSS Statistics 26.0 (IMB, USA). Results were exhibited as interquartile ranges (IQR) as well as median values. The comparison of quantitative data, which do not follow standard distribution between groups, was calculated using the Mann–Whitney U-Test, while Pearson Chi-square and Fisher exact tests were applied on nominal variables to determine associations among them. A *p*-value of <0.05 was scrutinized as statistically significant. The data was entered in SPSS and validated by an additional statistical analyst for reliability.

## 3. Results

Depending on bacteriological as well as intraoperative findings, there was an establishment of two patient groups (Table 1). The AcA group consisted of 49 patients (52.7%) with a positive culture sample from the peritoneal cavity whereas those with a negative culture were classified in the AsA group, this group consisting of 44 patients (47.3%). *E. coli* was identified in 79 patients (84.9%), thus it is the most common representative of appendiceal intraluminal microbiota in simple and complex appendicitis. *P. aeruginosa* was the most prevalent microorganism of the extraluminal appendiceal microbiota (AcA/AsA: 15/5).

A majority of the patients (76.9%), who had an inserted drainage tube, were diagnosed with AcA (*p* < 0.001) (Table 1). In a comparison between both groups, it was suggested that AuA had a slightly shorter median postoperative hospital stay, of five versus six days. The clinical characteristics of patients are shown in Table 1.

In Table 2, the number of the prevalent isolates per group is shown, AcA and AsA, respectively. A total of 25 different species were identified from samples obtained from patients with AsA, while 38 species were identified from samples of patients with AcA. The most commonly isolated isolate from the appendices were *E. coli*, which was found in 79 samples, followed by *P. aeruginosa* found in 20 samples, *Sphingomonas paucimobilis* (*S. paucimobilis*) in 9 samples, *Klebsiella pneumoniae* (*K. pneumoniae*) in 7 samples, *Bacterioides fragilis* (*B. fragilis*) in 5 samples and *Citrobacter braakii* (*C. braakii*) in 3 samples (Table 2).

Antibacterial susceptibility testing results are listed in Table 3. A total of 79 isolates of *E. coli* were identified and varied in antibacterial susceptibility. All strains were susceptible to meropenem and amikacin. Five (8.5%) strains were resistant to ceftazidime; thirty-two (54.2%) to ampicillin; six (10.2%) to cefotaxime; six (10.2%) to imipenem; eight (13.6%) to ciprofloxacin; six (10.2%) to chloramphenicol; two (3.4%) to ertapenem; eighteen (30.5%) to amoxicillin/clavulanic acid; one (1.7%) to piperacillin/tazobactam; and one (1.7%) to gentamicin. Additionally, five ESBL-producing strains of *E. coli* were also isolated.

*P. aeruginosa*, the second most common causative agent, showed a high prevalence in acute complicated appendicitis cases. A good response was shown during susceptibility testing to ceftazidime with only 26.3% of isolates being resistant. Ampicillin resistance was noted in 78.9% of isolates, while in 63.2% to cefotaxime, in 36.8% to imipenem, in 52.6% to chloramphenicol, in 10.5% to ciprofloxacin and piperacillin/tazobactam, in 63.2% to ertapenem and in 84.2% to amoxicillin/clavulanic acid. All tested strains were susceptible to meropenem, amikacin and gentamicin. Antibacterial susceptibility of other bacteria that were isolated in this study are shown in Table 3. *Citrobacter* spp. tested resistant to all antibiotics with the exception of amoxicillin/clavulanic acid, while *Klebsiella* spp. was resistant to cefotaxime, amikacin, gentamicin as well as chloramphenicol.

## 4. Discussion

The choice of the correct empirical antibacterial therapy is complex as it requires a clinician to decide on the most suitable antibiotic treatment prior to receiving the results of laboratory tests, isolation of the pathogen and detection of its antimicrobial susceptibility. Therefore, in order to produce an accurate algorithm for the most effective empirical treatment, it is crucial to have knowledge of the most common causative agents found in a specific geographical region, their profile of antimicrobial resistance and ability to develop resistance against the most frequently used antibiotics [4].

An argument exists regarding the prevalent causative agents, some authors consider *E. coli* and anaerobic *Clostridium perfringens* to be the most common [8], while others indicate *Klebsiella* spp., *Enterobacter* spp. [9], or *Bacteroides fragilis*, *P. aeruginosa*, *Enterococcus* spp. and alpha and gamma haemolytic streptococci as the most common [4].

Results of our research demonstrate that approximately 63% of *P. aeruginosa* isolates were resistant to cefotaxime, and about 26% against ceftazidime. *P. aeruginosa* has also been shown to be one of the most frequent causative agents of bacterial appendicitis. It has a high level of antibacterial resistance, which must be taken into account when devising the algorithm for empirical treatment [10]. Cefotaxime is included in the treatment algorithm of the Children’s Hospital for treatment of intraperitoneal infections in pediatric patients [11]. Although both cefotaxime and ceftazidime belong to third generation cephalosporins, ceftazidime has shown higher efficacy in the treatment of infections with Gram-negative bacteria, especially *Pseudomonas* spp., an important causative agent of nosocomial infections [12]. However, ceftazidime-resistant strains have been discovered. Resistance mechanisms against ceftazidime in *P. aeruginosa* include the production of beta-lactamase, encoded by genes acquired via horizontal gene transfer, or by increased production of a drug-induced, broad-spectrum, chromosomally encoded class C beta-lactamase with altered affinity [12]. According to research data, frequency of resistance against ceftazidime in *P. aeruginosa* isolates in Eastern European countries is approximately 26% [13], which greatly coincides with the findings of our study. Intrinsic antimicrobial resistance of *P. aeruginosa* also needs to be considered. Prescribing third generation cephalosporins, for example, ceftriaxone or antibiotics, such as sulbactam or ampicillin, would be futile, as these antibiotics will not be able to hinder the growth of these microorganisms [14].

Different hospitals use various treatment algorithms in the case of appendicitis. Ceftriaxone, if patients are not allergic to it, or ciprofloxacin, in case of allergy to cephalosporins, are frequently used. Metronidazole is also prescribed in addition to the aforementioned antibiotics [15]. Ceftriaxone belongs to third-generation cephalosporins and it has the following antibacterial spectrum: *Staphylococcus aureus* (methicillin susceptible), coagulase-negative *Staphylococci*, *Streptococcus pneumoniae* (penicillin susceptible), *Streptococcus* spp., *Haemophilus influenzae*, *Moraxella catarrhalis*, *Neisseria meningitidis*, *Neisseria gonorrhoeae*, *Enterobacteriaceae*, *E. coli. P. aeruginosa* was one of the common causative agents isolated in our study, however, the spectrum of ceftriaxone does not cover this microorganism, therefore cefotaxime, as well as ceftriaxone, would not be an appropriate choice for treatment of acute complex appendicitis [16]. Recent studies on antibacterial treatment of infections, caused by gut microbiota, have also demonstrated that the antibacterial activity and development of resistance to ceftriaxone does not differ from that of cefotaxime [17].

Our research reflects that *P. aeruginosa* was prevalent in samples obtained from patients with acute complex appendicitis. All strains were sensitive to meropenem, which inhibits cell wall synthesis and is not affected by beta-lactamase. Drusano et al. looked into the potential use of fosfomycin in the treatment of infections with *P. aeruginosa* and came across that the bacteria rapidly developed resistance against fosfomycin. Therefore, they suggested switching treatment from monotherapy to combination therapy with fosfomycin and meropenem. A synergistic effect was noticed with fosfomycin eradicating the meropenem-resistant mutants and meropenem working against fosfomycin-resistant strains. Thus, this combination could be endorsed as a treatment strategy for wider use in the future [18]. Another combination displaying encouraging results in research settings is meropenem in conjunction with ceftazidime [19].

In the past ten-year period, antibiotic combinations of cefatizidime/avibactam, ceftolozane/tazobactam and piperacillin/tazobactam have been explored as prospective treatment options [20].

Avibactam is an affiliate of the class of azabicycloalkanes. Avibactam is a non-beta-lactam beta-lactamase inhibitor that is accessible in combination with ceftazidime. This combination was approved by the Food and Drug Administration (FDA) on 25 February 2015 for the treatment of complex intra-abdominal infections in combination with metronidazole [21]. This combination has shown an efficacy of up to 90% against ceftazidime-resistant strains of *P. aeruginosa* [22]. Combined treatment with ceftazidime-avibactam and colistin has shown promise in treating infections with XDR (extremely drug-resistant) *P. aeruginosa* [23]. Levels of antimicrobial resistance to ceftazidime and avibactam across different regions do not show significant variability. They retain sufficient activity against Gram-negative bacteria, especially the *Enterobacteriaceae* family. *P. aeruginosa* is less susceptible to ceftazidime and avibactam compared to *Enterobacteriaceae*. Ceftazidime and avibactam cannot be used against microorganisms with intrinsic resistance. Strains that display resistance to ceftazidime and avibactam should be treated with other effective antimicrobials or in combination with other antibiotics [14].

Ceftolozane/tazobactam was accepted by the FDA in 2014, shortly before ceftazidime/avibactam was approved for the same indications. It is highly effective in combinations with meropenem and levofloxacin [20]. Nevertheless, antimicrobial resistance remains an issue with around 10% of *P. aeruginosa* strains displaying resistance to ceftolozane/tazobactam [24]. 

The combination of piperacillin/tazobactam includes an anti-pseudomonal penicillin and a beta-lactamase inhibitor. The mechanism of action is based on inhibition of biosynthesis of mucopeptides of the cell wall by binding to one or multiple penicillin-binding proteins. The antibiotic is highly effective during the growth or log stage [25,26]. Treatment protocols have extensive variations, yet most commonly include in-hospital treatment for one to two days (e.g., ceftriaxone/metronidazole, piperacillin/tazobactam or ciprofloxacin/metronidazole) until symptoms are resolved and the WBC count is normalized. This is followed by oral antibiotic therapy in the outpatient setting (e.g., amoxicillin/clavulanic acid or ciprofloxacin and metronidazole) [27].

Amikacin in our study demonstrated significant efficacy against isolates from the samples. It is a broad-spectrum semi-synthetic aminoglycoside antibiotic, derived from kanamycin with antimicrobial properties. Amikacin is bound irreversibly to the bacterial 30S ribosomal subunit, subsequently locking 16S rRNA and S12 protein within the 30S subunit. This leads to interference with the translational initiation complex and misreading of mRNA, thereby hampering protein synthesis and resulting in a bactericidal effect. This agent is usually used for short-term treatment of severe infections due to susceptibility of various strains of Gram-negative bacteria [28]. Data are scarce regarding amikacin-resistant *Pseudomonas* spp. Loho et al. showed that only two *P. aeruginosa* isolates were resistant against amikacin. Its amalgam with doripenem is synergistic and improves treatment results [29].

The most confined microorganism from our patients’ samples was *E. coli*, especially in those treated for acute simple appendicitis. This finding concurs with results obtained by other authors [4,8,30,31,32,33,34]. Our facts reveal that strains of *E. coli* are sensitive to antibacterial agents such as amikacin, and meropenem, which are in line with recent studies by other researchers [31]. Strains resistant to other antibacterial agents included in the treatment algorithms were also discovered in this study, such as cefotaxime and ceftazidime. In total, 6 strains of 49 were found to be resistant to cefotaxime and 5 strains to ceftazidime. Only five isolates (8.5%) were ESBL-positive. This falls in with the data from other studies determining the prevalence of ESBL-producing *E. coli* in Latvia. Data from the leading hospitals in Latvia showed a decline in the number of ESBL-producing *Enterobacteriaceae* in 2020 when compared with data collected in 2017. About 15–20% of *E. coli* isolates displayed ESBL activity [32].

To prevent the continued spread of infection in cases of acute appendicitis with complications, such as perforation, empirical treatment could include ceftriaxone (in combination with metronidazole) or ertapenem for children above the age of 1 month. Other options for empirical treatment could include piperacillin/tazobactam, imipenem or meropenem. The main aim of an appropriate antibacterial treatment regimen is to prevent complications associated with infection. Empirical antibiotic treatment should be based on information about the most commonly isolated microorganisms in a specified region and their profile of antibacterial resistance [34].

The purpose of using ceftazidime for non-surgical treatment of simple appendicitis is to limit bacterial growth associated with *P. aeruginosa* within the appendix, in order to prevent the destruction of the appendiceal wall and its subsequent perforation. Further research is necessary to determine what practical implications the findings of our study have.

The study had some limitations and deserves a word. Although the study was conducted in one of the biggest tertiary children’s hospitals in the country, we faced a limited number of resources in the odd hours, courier services, microbiological laboratory working hours, the COVID-19 pandemic and, lastly, in the beginning, the ethics committees’ non-approval for children below the age of seven years.

## 5. Conclusions

*E. coli* is the main causative agent of acute appendicitis in children demonstrating susceptibility to various antibiotics. *P. aeruginosa* is identified more frequently in cases of acute complex appendicitis compared to cases of acute simple appendicitis. *P. aeruginosa* is susceptible to agents of the cephalosporin group, such as ceftazidime, however, *P. aeruginosa* has phenotypic resistance to cefotaxime, which was also confirmed in our study. Therefore, cefotaxime should be removed from the guidelines for empirical treatment of acute appendicitis. Antibiotics with distinct implementation should be recommended for the treatment of acute complex appendicitis to prevent the development of antimicrobial resistance.

## Figures and Tables

**Table 1:** Overview of study population.

	AcA	AsA	Total	*p*-Value
Children, *n* (%)	49 (52.7)	44 (47.3)	93	0.269
Age, median (IQR)	12 (9–14)	13 (10–15)	-	0.194
Laboratory values, median (IQR)				
WBC count (×10^9^/L),	17.01 (13.75–20.25)	14.79 (13.20–16.76)	-	0.019
CRP (g/L),	25.93 (4.50–89.68)	15.82 (2.86–39.29)	-	0.201
Neu	84.50 (80.93–87.00)	80.80 (73.90–84.80)		0.012
Alvarado Score, points, median (IQR)	8 (7–9)	7 (6–9)	-	0.098
Type of surgery, *n* (%)				
Laparotomy	7 (63.6)	4 (36.4)	11	
Laparoscopy	42 (51.2)	40 (48.8)	82	0.439
Drainage tube, *n* (%)	30 (76.9)	9 (23.1)	39	<0.001
Length of hospital stay, days, median (IQR)	6 (4–9)	5 (4–6)	-	0.002

AsA = acute simple appendicitis, AcA = acute complex appendicitis, WBC = White Blood Cells, CRP = C-Reactive Protein. Median values are presented with IQR (25%, 75%).

**Table 2:** Bacterial profile in acute complex appendicitis and acute simple appendicitis.

Bacteria	AcA 49 Patients		AsA 44 Patients		Total 93 Patients		
	No. of Patients with Respective Bacteria	% of Patients with Respective Bacteria	No. of Patients with Respective Bacteria	% of Patients with Respective Bacteria	No. of Patients with Respective Bacteria	% of Patients with Respective Bacteria	*p*-Value
*E. coli*	43	82.7	36	80.0	79	81.4	0.424 ^#^
*P. aeruginosa*	15	28.8	5	11.4	20	21.6	0.024 ^#^
*S. paucimobilis*	8	15.4	1	2.2	9	9.3	0.033 *
*K. pneumoniae*	3	5.8	4	8.9	7	7.2	0.704 *
*B. fragilis*	2	3.8	3	6.7	5	5.2	0.655 *
*C. braakii*	0	0	3	6.7	3	3.1	0.102 *

AcA = acute complex appendicitis, AsA = acute simple appendicitis, ^#^—Pearson Chi-square test, *—Fisher Exact test.

**Table 3:** Antimicrobial resistance and susceptibility of isolated pathogens.

	*E. coli n*, %		*P. aeruginosa n*, %		*Klebsiella n*, %		*Citrobacter n*, %	
	R	S	R	S	R	S	R	S
CAZ	5	54	5	14	1	8		5
	8.5	91.5	26.3	73.7	11.1	88.9		100.0
AMP	32	27	15	4	7	2		5
	54.2	45.8	78.9	21.1	77.8	22.2		100.0
CTX	6	53	12	7		9		5
	10.2	89.8	63.2	36.8		100.0		100.0
MRP		59		19	1	8		5
		100.0		100.0	11.1	88.9		100.0
IMI	6	53	7	12	1	8		5
	10.2	89.8	36.8	63.2	11.1	88.9		100.0
AK		59		19		9		5
		100.0		100.0		100.0		100.0
CN	1	58		19		9		5
	1.7	98.3		100.0		100.0		100.0
CIP	8	51	2	17	1	8		5
	13.6	86.4	10.5	89.5	11.1	88.9		100.0
C	6	53	10	9		9		5
	10.2	89.8	52.6	47.4		100.0		100.0
ETP	2	57	12	7	1	8		5
	3.4	96.6	63.2	36.8	11.1	88.9		100.0
AUG	18	41	16	3	2	7	5	
	30.5	69.5	84.2	15.8	22.2	77.8	100.0	
TZP	1	58	2	17	1	8		5
	1.7	98.3	10.5	89.5	11.1	88.9		100.0

Abbreviations: CAZ—ceftazidime, AMP—ampicillin, CTX—cefotaxime, MRP—meropenem, IMI—imipenem, AK—amikacin, CN—gentamicin, CIP—ciprofloxacin, C—chloramphenicol, ETP—ertapenem, AUG—amoxicillin/clavulanic acid, TZP—piperacillin/tazobactam.
